# Poly-beta-amino-esters nano-vehicles based drug delivery system for cartilage

**DOI:** 10.1016/j.nano.2016.10.001

**Published:** 2017-02

**Authors:** Stefano Perni, Polina Prokopovich

**Affiliations:** School of Pharmacy and Pharmaceutical Sciences, Cardiff University, Cardiff, UK

**Keywords:** Cartilage, Dexamethasone, PBAE, OA

## Abstract

The efficient delivery of therapeutic molecules to the cartilage of joints is a major obstacle in developing useful therapeutic interventions; hence, a targeted drug delivery system for this tissue is critical. We have overcome the challenge by developing a system that employs electrostatic attraction between the negatively charged constituents of cartilage and a positively charged polymer, poly-beta amino esters (PBAEs). We have demonstrated cartilage uptake of dexamethasone (DEX) covalently bound to the PBAE was doubled and retention in tissues prolonged compared to the equivalent dose of the commercial drug formulation. Moreover, no adverse effects on chondrocytes were found. Our data also show that PBAEs can bind not only healthy cartilage tissues but also enzymatically treated cartilage mimicking early stages of OA. Our PBAEs-prodrug technology's advantages are fourfold; the specificity and efficacy of its targeting mechanism for cartilage, the ease of its production and the low-cost nature of the delivery system.

## Graphical Abstract

We have overcome the challenge delivering drugs into cartilage by developing a system that employs electrostatic attraction between the negatively charged constituents of cartilage and positively charged poly-beta amino esters. We have demonstrated that cartilage uptake of Dexamethasone was doubled and retention in tissues prolonged compared to the equivalent dose of the commercial drug formulation when bound to PBAE. Moreover, no adverse effects on chondrocytes were found.

**Figure:**
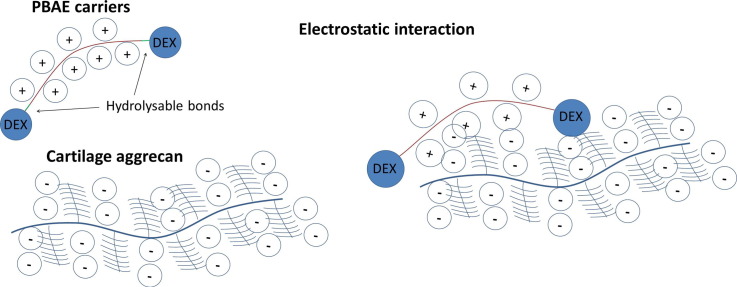

Cartilage is the connective tissue that constitutes the load-bearing surfaces of synovial joints; they allow the synovial joints' low friction and pain-free movements.^1^ The structure of the extracellular matrix of cartilage is characterized by the presence of glycosaminoglycans (GAGs). The resulting structure is highly negatively charged allowing cartilage to perform the required functions of shock absorber and low friction surface.

Osteoarthritis (OA) is the most common disease affecting joint in the USA, with a reported 10% and 13% of male and female population, respectively, over 60 years old experiencing symptomatic OA,^2^ similar incidence was found also in the UK,^3^ with about 8.7 millions of patients.^4^ Some of the most common predictors of OA are obesity and age^5^, ^6^; therefore, the number of OA diagnoses is expected to increase in light of the aging population along with growing obesity.^4^ At present, therapies capable of providing only a short term relief of pain and inflammation are offered; these can be through the administration of steroidal or non-steroidal anti-inflammatory drugs (NSAID). Many of the drugs used to treat arthritis have serious side effects, i.e. dexamethasone (DEX) has been linked to bone loss, muscle weakness and atrophy, suppression of the adrenal gland, increased risk of infections, peptic ulcer disease and growth retardation.^8^ Moreover, drug penetration and retention in cartilage is minimal.^9^ Hence, the development of a delivery system capable of increasing the partitioning of steroids between the cartilage tissue and synovial fluid would reduce the amount of drug dispersed, and consequently, the incidence of side effects. Many different approaches have been developed to improve intra-articular treatment of joints i.e. PLGA^10^ and chitosan^11^ based microcrospheres; self-assembling nanoparticles,^12^ liposomes^13^ and calcitonin based nanocomplexes^14^ and polypeptides.^7^, ^15^, ^16^, ^17^ However, none of these approaches is ideal; therefore, new approaches are needed to effectively tackle the problem.

Poly-beta amino esters (PBAEs) are a class of molecules obtained from the co-polymerization of diacrylate and amine molecules^18^; these molecules possess positive charges and are biodegradable; they have been the subject of numerous studies employing them as DNA delivery systems.^19^, ^20^, ^21^, ^22^, ^23^, ^24^ The biocompatibility and biodegradability of PBAEs is the main benefit of these compounds compared to other available positively charged polymers (poly-cations) such as: poly-L-lysine and polyethylenimine (PEI).^19^, ^20^, ^21^, ^22^, ^23^, ^24^ The cytocompatibility of PBAEs has also been demonstrated both in vitro^25^, ^26^ and in vivo.^27^, ^28^

We hypothesize that positively charged PBAE (Figure 1, *A*). could be employed as a drug delivery system to overcome the challenge of delivering drugs to cartilage exploiting the electrostatic attraction toward the negatively charged glycosaminoglycan (sGAG) components of the cartilage extracellular matrix (Figure 1, *A*). The main objectives of this study were: (a) to demonstrate the cartilage penetrating properties of PBAE; (b) to determine uptake/retention enhancement in cartilage of a OA model drug (DEX) when either covalently bound to both ends of the PBAEs chains after functionalization or electrostatically linked to the PBAE chain (mixing the drug with PBAEs); (c) to establish efficacy in GAG depleted cartilages representing OA and (d) to establish PBAE cytocompatibility toward chondrocytes.

## Methods

A two-steps process was employed to synthesized end-functionalized polymers. First, acrylate-terminated polymers were prepared (Figure A1), then ethylenediamine was conjugated at both ends of the chains (Figure A2) in order to provide a moiety for subsequent drug conjugation (Figure A4).

### Polymer synthesis

Piperazine or 4,4′-Trimethylenedipiperidine (Sigma, UK) were used as amine monomers. Acrylate-terminated poly(β-amino ester)s were synthesized by mixing 1,4 butanediol diacrylate (Sigma, UK) and amine monomers in a 1.1:1 ratio in Dichloro-methane (DCM) (Fischer, UK) at a concentration of 5 ml of DCM each 3.7 mmol of acrylate. The polymerization was then performed under stirring at 50 °C for 48 h. PBAEs were precipitated through pouring the reaction mixture in about 10 times the volume of diethyl-ether (Fischer, UK) under vigorous mixing; the solvent was removed under vacuum.

Acrylate-terminated polymers were dissolved in DCM at a concentration of 31.13% w/w; whilst ethylenediamine was dissolved in DCM to a concentration of 0.25 mol/l. The capping reaction (Figure A2) was performed by mixing the polymer/DCM solution with ethylenediamine solution at a ratio of 800 μl per 321 mg of polymer solution; the mixture was kept for 24 h at room temperature under mixing.

End-capped PBAEs (amino terminated) were recovered through precipitation in diethylether under vigorous mixing, the unreacted amine were removed centrifuging the suspension of PBAE in diethyl-ether for 2 min at 1155 g. The supernatant was removed and the PBAEs washed twice with diethylether. The amino end-capped PBAEs were then dried under vacuum.

PBAE made using piperazine and 4,4′-trimethylendipiperidine will be denoted as A1 and A2 through the text, respectively.

### Dexamethasone succinylation

Dexamethasone (DEX) was succinylated mixing 200 mg of drug with 200 mg of succinic anhydride and 10 mg of 4-(Dimethylamino)pyridine (DMAP) in 50 ml Dimethyl-formamide (DMF) (Figure A3). The reaction was performed under nitrogen for 24 h at room temperature with mixing. The solvent was removed under vacuum (in a rotary evaporator) and the solid residue of succinylated Dexamethasone (DEX-succ) was purified through repeated washing with dH_2_O. Finally, the product was dried under vacuum.

### Conjugation of DEX to PBAEs

The conjugation of DEX-succ was performed mixing 80 mg of amine end-capped PBAE with 8 mg of succinylated drug, 8 mg of N,N′-Dicyclohexylcarbodiimide (DCC) and 8 mg of N-hydroxysulfosuccinimide (NHS) in 25 ml of DCM (Figure A4). Conjugates PBAEs-drug were precipitated through pouring the reaction mixture in about 10 times the volume of diethyl-ether under vigorous mixing; the solvent was removed under vacuum. PBAEs-drug conjugates were washed twice with diethyl-ether. The final product was then dried under vacuum.

### PBAE characterization

Organic phase gel permeation chromatography (GPC) was performed in a PL-GPC 20 (Polymer Laboratory) system equipped with two ResiPore columns in series using THF as eluent at a flow rate of 1.0 ml/min. The refractive index detector and columns were maintained at room temperature throughout the runs. 100 μl of each sample prepared at 10 mg/ml in THF were injected, and each sample was given 30 min to elute from the column. The molecular weights (Mw and Mn) of the polymers were reported relative to mono-disperse polystyrene standards (EasiCal, Agilent).

PBAE sizes were measured using Dynamic Light Scattering (DLS) using a Malvern Zetasizer Nano ZS (Malvern Instruments, Malvern, U.K.); average electrophoretic motilities were measured at 25 °C also using Malvern Zetasizer Nano ZS (Malvern Instruments, Malvern, U.K.) and zeta potentials were calculated using the Smoluchowsky model. For both analysis, un-capped (acrylated terminated) PBAE were dissolved in 100 mM phosphate buffer (pH = 6.0) at about 20 mg/ml.

### Cartilage samples

Bovine steers immature (7-day-old) feet were obtained from a local abattoirs. Articular cartilage explants were surgically removed under sterile conditions from metacarpo-phalangeal joints. Full depth explants were excised using a 6 mm diameter biopsy punches from the medial aspect of the medial condyle of individual joints. Explants were placed initially in Dulbecco's modified Eagles medium (DMEM; Invitrogen, Paisley, UK) and washed in the same medium to remove blood and small particulates due to the presence of a small amount of subchondral bone lining the basal aspect of cartilage.

GAG depleted samples were obtained digesting the samples in a solution of trypsin 1 mg/ml in PBS for 24 h at 37 °C and washed three times in fresh PBS.

### Cartilage GAG content quantification

The amount of GAG present in the cartilage samples before and after depletion was determined through the DMMB (Dimethyl-Methylene Blue) assay.^29^

### DEX uptake into cartilage using PBAE-DEX

A PTFE transport chamber was designed and manufactured to study one-way diffusion of solutes entering into cartilage (Figure 2, *A*); the chamber walls were treated with casein to block non-specific binding of solutes to PTFE surfaces. Cartilage disks (6 mm diameter, ~0.4 mm thick) were cut in half, weighted and placed in one of the holding slots machined into the chamber. The chamber facing the superficial zone was filled with 50 μl of a known concentration of PBAEs-drug formulation in PBS supplemented with protease inhibitors; the other chamber side was filled with 50 μl of PBS containing protease inhibitors alone. The chamber was then placed in a Petri dish containing dH_2_O and covered to minimize evaporation then placed inside an incubator at 37 °C; stagnant layers at cartilage surfaces were prevented placing the dish on a slow-speed rocker. At required intervals a sample was removed, washed in copious amount of water and placed in an Eppendorf containing 1 ml of digestion buffer. Experiments were performed on duplicate samples originated from 3 different animals.

Comparison of the drug uptake was performed between a solution of Dexamethasone phosphate (DEX-P) at the advised concentration of 4.4 mg/ml, equivalent to 4 mg/ml of DEX, and a solution of PBAE-DEX containing the same amount of steroidal drug.

### Cartilage digestion

Cartilage samples were digested using a phosphate buffer 0.2 M at pH = 6.8 containing 300 mg/l of papain, EDTA 1 mM and Dithiothreitol (DTT) 2 mM. Samples were placed in 1 ml of the digestion buffer and incubated at 50 °C for 24 h.

### DEX retention

Cartilage samples were exposed to the DEX solution in the apparatus described above for 10 min and after washing in copious amount of water, they were place in an Eppendorf containing 0.5 ml of PBS. Samples were stored for up to 2.5 h at 37 °C, at fixed intervals the cartilage was removed from the PBS solution, washed with water and placed in 1 ml of digestion buffer.

### DEX quantification

Dexamethasone in the digestion buffer was quantified through reverse phase-HPLC. An Agilent series 1100 HPLC system was equipped with a TeknoKroma TRACE EXCEL 120 ODSB 5 μm analytical column thermostated at 25 °C. The injection volume was 25 μl, the mobile phase was PBS:acetonitrile:glacial acetic acid 70:26:4 with a flow rate of 1 ml/min and the detector was a UV spectrophotometer at 244 nm. The amount of drug present in the cartilage was then expressed as mass of drug per cartilage mass.

### Viability of chondrocytes after exposure to PBAE-DEX

The cartilage samples underwent a 1 h pre-digestion stage at 37 °C with 0.4% w/v pronase (Sigma, UK), dissolved in 4-(2-hydroxyethyl)-1-piperazineethanesulfonic acid (HEPES)-buffered DMEM (Gibco, UK) supplemented with 50 U/50 μg/ml penicillin–streptomycin (Sigma Aldrich) and 2.5 mg/l amphotericin B. Cartilage samples were placed in sterile Eppendorfs containing the filter-sterilized enzyme solution by passage through a 0.22-μm filter at a concentration of 1 ml solution for 5 cartilage extracts. The cartilage fragments were then washed twice with sterile PBS. 1 ml of collagenase II (Sigma, UK) 0.1% w/v solution was added to 5 cartilage samples contained in an Eppendorf. Collagenase II was prepared in 4-(2-hydroxyethyl)-1-piperazineethanesulfonic acid (HEPES)-buffered DMEM (Gibco, UK) supplemented with 50 U/50 μg/ml penicillin–streptomycin (Sigma Aldrich) and filter sterilized. Samples were incubated for 16 h at 37 °C.

The triturate suspension was passed through a 100-mm nylon cell strainer (Fisher, UK) to remove matrix debris, and added to 10 ml DMEM with GlutaMAX (DMEM/F-12) (Gibco, UK) media, supplemented with 20% (v/v) FBS, 50 U/50 μg/ml penicillin–streptomycin (Sigma Aldrich) and 2.5 mg/l amphotericin B. Chondrocytes were centrifuged at 1500 rpm at 4 °C for 10 min to pellet the cells. The supernatant was discharged and the pelleted cells were washed in sterile PBS. After centrifugation at 1500 rpm at 4 °C for 10 min, the cells were suspended in supplemented 10 ml of DMEM/F-12 (10% FBS) and aseptically transferred in cell culture flasks. Chondrocytes were cultured in a humidified incubator at 37 °C and 5% CO_2_. After 3 days chondrocytes were washed in sterile PBS and trypsinated. 24 well plates were inoculated with approximately 6000 cells/well in 1 ml of DMEM/F-12 supplemented with 10% FBS and 1% penicillin–streptomycin. Chondrocytes were grown for 2 days in a humidified incubator at 37 °C and 5% CO_2_ before 15 μl of a solution containing 4 mg/ml DEX was added. The DEX solutions were prepared using either Dexamethasone phosphate (DEX-P) or PBAE-DEX conjugates.

24 well plates were incubated in a humidified incubator at 37 °C and 5% CO_2_ for up to 3 days.

Chondrocytes viability was assessed through MTT and LDH assay kits (Sigma, UK) according to manufacturer's protocols. Experiments were performed in triplicate on cells originated from 3 different animals.

### Determination of diffusion coefficient of PBAE in cartilage

Amino terminated PBAEs were fluorescein-tagged (PBAE-FITC) using FluoroTag™ FITC Conjugation Kit (Sigma, UK) according to manufacturer's recommendations.

The diffusion coefficients of PBAE were determined using the same PTFE transport chamber and arrangements described in Figure 2. The chamber facing the superficial zone was filled with 50 μl of a known concentration of PBAEs-FITC supplemented with protease inhibitors; the other chamber side was filled with 50 μl of PBS containing protease inhibitors alone. After diffusion for a set length of time, 33 μl of liquid for both chamber were removed and added to 67 μl of fresh PBS contained in black 96 wells plate. Fluorescence was read using FLUOstar OPTIMA Microplate Reader (BMG Labtech, UK) with Ex =480 nm and Em = 520 nm. The parameters were set assuring that the intensity response was in the linear range of concentrations as determined during PBAE-FITC purification.

The ratio of fluorescence between the two sides of the cartilage was plotted against diffusion time (*t*) and fitted with the following equation using an in-house written FORTRAN code, in order to identify the “break-through time” (*t*_*lag*_);

(1) $$\begin{array}{c} \mathrm{Ratio fluorescence}\\ \mathrm{between cartilage sides} \end{array}\;\left(t\right)=\left\{\begin{array}{cc} \left({t-t}_{\mathrm{lag}}\right)*K & {\;t>t}_{\mathrm{lag}}\\ 0 & \;{t<t}_{\mathrm{lag}} \end{array}\right)\;$$

Where *K* is related to PBAE steady state flux. Then, the diffusion coefficient (D) was calculated as^30^:

(2) $$D=\frac{\delta^{2}}{6\;t_{\mathrm{lag}}}$$

where:

*δ* is the cartilage sample thickness.

Experiments were performed on triplicate samples originated from 3 different animals (for a total of 9 measurements).

### Fluorescent imaging of cartilage

Cartilage samples were removed from the perfusion chamber after 1 min of exposure to FITC tagged PBAEs and rinsed in PBS. The protocol employed to qualitatively assess the penetration of PBAE in cartilages is shown in Figure A6; a radial strip about 0.5 mm thick was cut from the cartilages half disks exposing the middle section of the sample. The cartilage sections were placed on a microscope glass slide and imaged using a Leica DM, IRB microscope.

### DEX release from PBAE-DEX

Release kinetics of DEX from PBAE-DEX were determined using dialysis membranes (GE Healthcare Mini Dialysis Kits - 1 kDa cut-off). A solution of PBAE-DEX in PBS (4 mg of DEX/ml) was placed in the Dialysis Kits and immersed in PBS and incubated at 37 °C for up to 48 h under mixing; at prefixed intervals the concentration of DEX released in the solution was quantified.

The percent of DEX released from PBAE-DEX after a set time (*t*) was calculated as:

(3) $$\mathrm{DEX}\;\mathrm{release}\;\left(t\right)\left[\%\right]=\frac{\mathrm{conc}.\mathrm{DEX}\;\left(t\right)*\mathrm{Vol}}{\mathrm{mass}\;\mathrm{DEX}\;\left(t=0\right)}*100$$

where:

*conc. DEX*: is the concentration of DEX in the dialysis fluid;
*Vol*: is the volume of the dialysis fluid;
*mass DEX (t* *=* *0)*: is the initial mass of DEX in PBAE-DEX.

### DEX uptake into cartilage using DEX mixed with PBAE

A solution containing 4.4 mg/ml of Dexamethasone phosphate (equivalent to 4 mg/ml of Dexamethasone) and the same quantity of pure end-capped PBAE that would be found in PBAE-DEX was prepared and employed in the cartilage uptake experiments previously described.

### Statistical analysis

Chemical physical properties and diffusion coefficients of PBAE date were compared using *t* test with a level of significance of 0.05. Drug uptake, release and chondrocytes viability were analyzed using one-way ANOVA to determine any significant difference between the mean values, this was followed by Tukey's *post-hoc* test (*p* < 0.05). Statistical analysis was performed using SPSS.

## Results

The characteristics of both A1 and A2 are reported in Table 1. Both PBAE had a positive charge, +11.60 and +8.94 mV respectively; A1 charge was statistically significantly higher than A2 (*p* < 0.05). Examples of PBAE size distribution measured through Dynamic Light Scattering (DLS) are shown in Figure A7; the size of A1 in buffer pH = 6 was 286 nm and greater than A2 (p < 0.05) that was 153 nm. No difference in both the weighted average molecular mass (Mw) and the numerical average molecular mass (Mn), estimated through GPC, was noticed between A1 and A2 with values of 12 kDa for Mw and 7 kDa for Mn.

Exposure to trypsin resulted in a reduction of 54 ± 9% of the GAG content of the cartilage samples.

Fluoro-tagging of PBAE with FITC revealed that PBAE were able to penetrate the cartilage tissues used (Figure 3) in about 1 min; it also appeared that A1 penetrates cartilage easier than A2 as little fluorescent dye was noticed in the cartilage sample far from the surface exposed to PBAE. The effective diffusion coefficient (D) depends on the structure of the PBAE and is connected to the time necessary to the PBAE to cross the full thickness of the sample (t_lag_) as shown in (Figure 3); in cartilage samples with original level of GAG, the diffusion coefficient for A1 ($D_{A1})$ was greater than for A2 ($D_{A2})$ (*p* < 0.05), 8 × 10^−6^ cm^2^/s and 5.5 × 10^−6^ cm^2^/s respectively. Moreover, the diffusion coefficient in GAG depleted cartilage for both PBAE was 1.3 × 10^−5^ cm^2^/s (*p* > 0.05) hence greater than the corresponding diffusion coefficient in cartilage with normal levels of GAG.

When cartilage samples were exposed to the same concentration of DEX, either as the commercial formulation of DEX-P or in the conjugated form to PBAE, the amount of drug present in the cartilage tissue (both normal and GAG depleted) increased monotonically with time; furthermore after each time point, conjugation of Dexamethasone to PBAE resulted in a higher amount of the drug (*p* < 0.05) in the cartilage (Figure 4, *A*) even after a very short period of time (1 min). The use of A2–1-DEX resulted in the concentration of DEX in the cartilage to increase only in the first minute and then remained almost constant (*p* > 0.05). Despite relying on the electrostatic attraction between the positive charges of PBAE and negative charges of the GAG molecules to deliver the drug in the cartilage, PBAE were also effective on GAG depleted cartilage (Figure 4, *B*) as the amount of drug recovered in the tissues after exposure to solutions of equal concentration of DEX was higher in case of both PBAE-DEX tested than DEX-P (*p* < 0.05).

DEX retention in cartilages was poor as the great majority of the drug was released from the cartilage in the first 30 min post uptake (Figure 4), C and D; in normal GAG sample DEX concentration fell below detection limit after 2.5 h (Figure 4, *C*) in case of DEX-P; when A1-DEX were employed DEX was still detectable in the cartilage even after 2.5 h, this was not the case for A2-DEX as no drug was detected in the samples after 90 min of release. In GAG depleted cartilage (Figure 4, *D*) the release of DEX-P was quicker than for normal GAG samples as the drug was not detected after 2.0 h when, furthermore the amount of DEX remaining in the tissue delivered through A1-DEX or A2-DEX was always higher than for DEX-P (*p* < 0.05).

The viability of cartilage cells (chondrocytes) was not affected after exposure to PBAE for at least 3 days (Figure 5). Cell viability was assessed with two different protocols (LDH and MTT) and both enzyme assays gave the same viability for samples exposed to DEX-P and samples exposed to PBAE-DEX (*p* > 0.05).

The ester bond conjugating DEX to both PBAE is almost completely hydrolyzed over a period of 2 days (Figure 6), with the majority of drug been released in the first few hours after contact with aqueous solution. The kinetic of release from A2 appeared slightly slower than that from A1.

When pure PBAEs were present along with DEX, but the two compounds were simply mixed together and not conjugated, the uptake of DEX was always lower than the corresponding case of pure steroidal drug DEX-P (Figure 7).

## Discussion

PBAEs are a class of polymers developed in the last two decades almost exclusively for DNA delivery. The vast application of PBAE to DNA delivery is based on the positive charge of PBAE that allows them to bind the negatively charged strands of DNA before entering the cells were the DNA is released and the PBAE hydrolyzed into biocompatible products.^18^, ^19^, ^20^, ^21^, ^22^, ^23^, ^24^ Effective and targeted delivery of drugs into cartilage is notoriously difficult because of the blood vessels absence and the tissue structure characterized by highly concentrated negatively charged proteoglycans. We assumed that PBAE positive charge could also be exploited for delivering drugs into cartilages through the electrostatic interaction with the proteoglycans and we demonstrated the feasibility of this technology.

**Figure 1:**
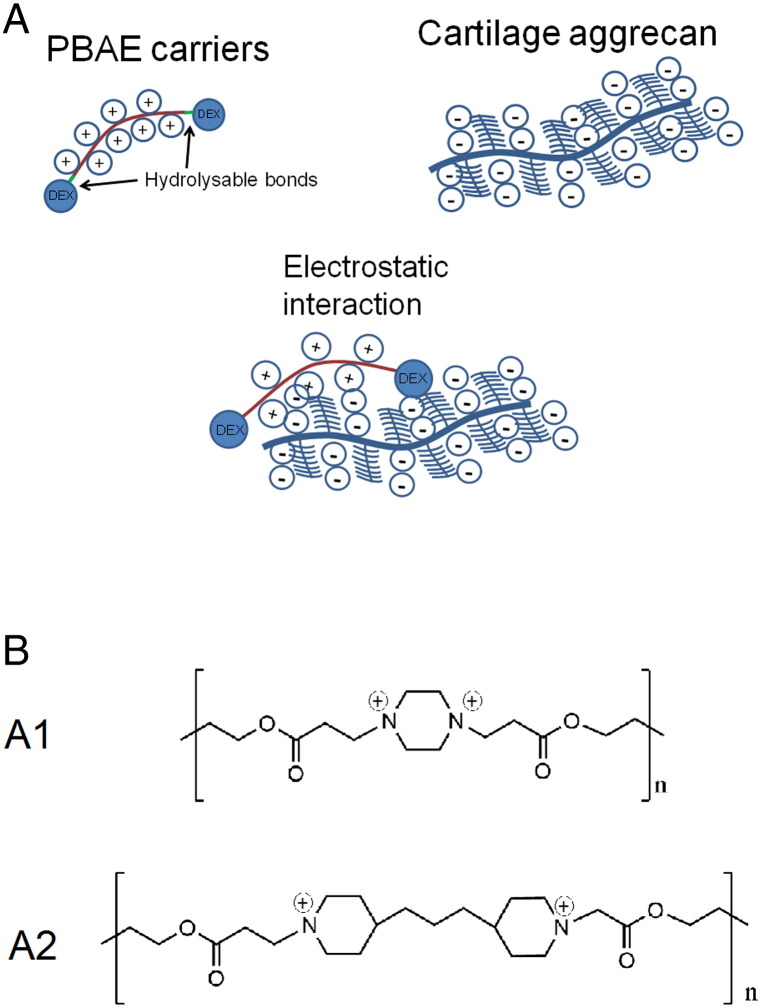
Pictorial schematization of the drug delivery mechanism developed **(A)**; structure of ionized PBAEs **(B)**.

**Figure 2:**
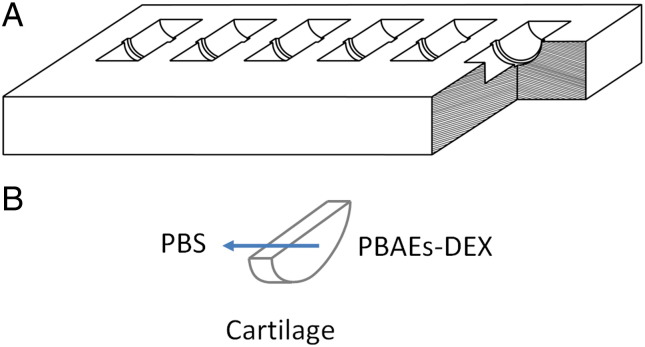
Scheme of the transport chamber **(A)** and of the flow through the cartilage sample **(B)**.

Both the molecular weights (Mw and Mn) and size of A1 and A2 found (Table 1) are similar to values reported in literature for poly-beta-amino-esters.^31^, ^32^ Instead, zeta potentials (Table 1) are lower than those reported for these polymers by Sunshine et al (2012),^31^ a possible explanation for the higher potential in this work is that their measurements were performed at pH = 5 instead of pH = 6 as used here; it is well known that fewer nitrogen are protonated at higher pH and, therefore, the polyelectrolyte exhibits lower zeta potential. This hypothesis is also substantiated by the finding of Kim et al (2014)^32^ that reported zeta potential of PBAE of about +13 mV when measured in PBS. Moreover, the difference in zeta potential observed between A1 and A2 can be attributed to the different amines used in the polymeric chains (Figure 1, *B*) because piperazine has pKb = 4.2 while piperidine has pKb = 2.9. The size of the two polymers when in suspension does not depend only on the chain length but also on the self-arrangement of the chains; in case of PBAE, it can be influenced by the pH of the solution through the protonation of the nitrogen.^33^

Counterions present in a solution mediate electrostatic interactions between charged molecules through the formation of electric double layers that screen the original charge present on the molecule thus the higher the concentration of counterions the lower the electrostatic forces; in order to account for this effect we employed PBS solution that mimic the ionic strength of biological fluids.

Trypsin is proteolic enzyme that is used to reduce the quantity of GAG presents in cartilage and such its use mimics osteoarthritis onset.^7^, ^35^, ^36^, ^37^, ^38^ The GAG reduction observed in our samples (about 50%) was in line with the outcome of similar treatments.^38^ The enzyme is dissolved in either PBS^38^ or Na_3_PO_4_^7^, ^36^ and it has been suggested that the GAG depletion outcome is independent from the choice of these solutions.^39^

In the delivery system presented in this work, PBAEs rely on their electrostatic attraction toward the negatively charged GAG to enhance drug uptake; therefore, a reduction in GAG could have resulted in lower efficacy of the drug delivery system in situations (cartilage affected by OA) when drugs are more likely to be needed. Our results demonstrated that, despite the reduction of GAG content, the uptake of DEX through PBAE-DEX was greater than in case of cartilage with normal amount of GAG (Figure 4). Similarly to these results, Elsaid et al (2013)^40^ found that cartilage GAG depletion enhanced drug-loaded micelle penetration into the tissues; Torzilli et al (1997)^41^ also reported diffusion of insulin and dextran to be inversely proportional the amount of proteoglycan cartilage content. Such phenomenon is likely to depend on the reduced steric inherence exhibited by GAG depleted cartilage to the penetration of PBAE as it has been shown that GAG depleted tissues exhibit higher hydraulic permeability^35^, ^36^ than normal cartilage tissue. This hypothesis is also confirmed by the higher diffusion coefficients of A1 and A2 in GAG depleted cartilage. Hence, PBAE is an effective drug delivery system for cartilage even when the amount of GAG is severely limited that more closely represent the situation when treatment is required like in OA affected patients.

**Figure 3:**
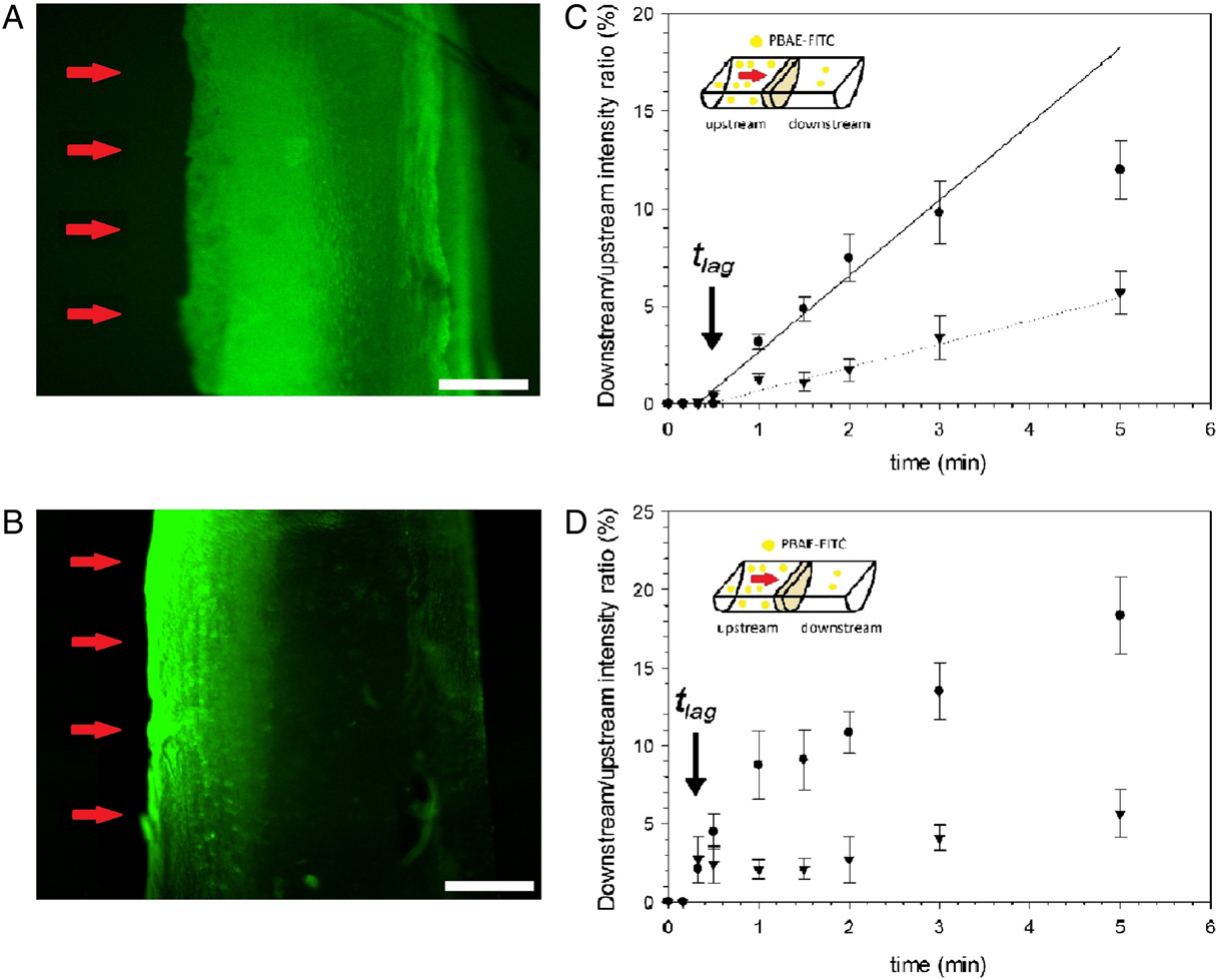
Examples of Epifluorescent images of normal cartilages exposed to **(A)** A1-FITC and **(B)** A2-FITC for 1 min in the perfusion chamber with flow direction marked by red arrows. Bar represent 150 μm. Non-equilibrium diffusive transport of PBAE-FITC across a 6 mm diameter, 400 μm thick normal **(C)** and GAG depleted **(D)** cartilage explants, plotted as the ratio between measured downstream and upstream concentration vs. time. Symbols (● A1 ▼ A2) line model fitting for t_lag_ determination.

We utilized the ability of FITC to bind primary amines to fluorescently tag the extremities of end-capped PBAEs as ethylenediamine provided such moiety. Our results, using PBAE tagged with FITC, provided evidence of PBAE penetrating through the cartilage tissue and not simply electrostatically bind to the surface of the cartilage (Figure 3). The lower diffusion coefficient measured for A2 compared to A1 (Figure 3) was consistent with the images of cartilages obtained using FITC tagged PBAE (Figure 3) and likely linked to the different amine compounds present in the PBAE structure.

**Figure 4:**
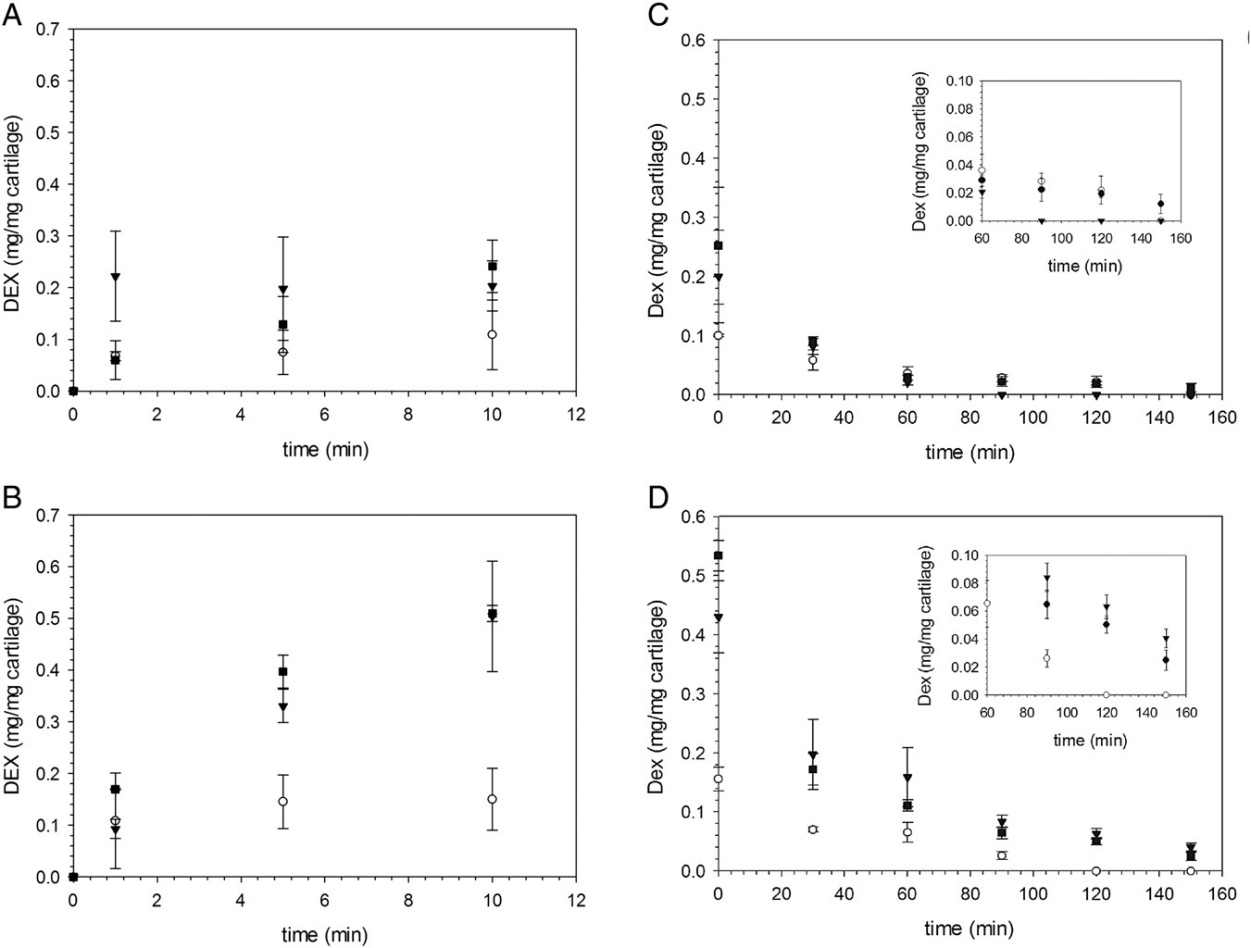
Comparison of DEX uptake in normal cartilage **(A)** and GAG depleted cartilage **(B)** using PBAE-DEX. Comparison of DEX retention in normal cartilage **(C)** and GAG depleted cartilage **(D)** using PBAE-DEX after uptake for 10 min. Insets highlight behavior after long periods of drug release. ■ A1-DEX ▼ A2-DEX ◯ DEX-P.

The size of the drug carrier directly impacts its cartilage penetration ability^41^, ^42^; it has been shown that the exclusion size for avidin in cartilage is about 10 nm, ^7^ analogously Elsaid et al (2013)^40^ gave evidence that 10 nm liposome were more effective than 100 nm micelle in delivery drugs into cartilage. The remarkable cartilage penetration ability exhibited by PBAE is despite their relative large size, that is in the order of hundreds of nanometers (Figure A6, Table 1); only few successful delivery systems for cartilage have sizes comparable to PBAE.^12^, ^13^ However, despite its pivotal role, size is not the only parameters affecting cartilage penetration, for example charge^7^ and surface modification^40^ have also been implicated in regulating cartilage diffusion. Our results also highlight the likely impact of the polymer structure as A2–1 is smaller than A1–1 but returns worst uptake and retention performance (Figure 4).

The release kinetic of DEX from PBAE-DEX (Figure 6) is dependent on the hydrolysis of the ester bond linking the steroidal drug to PBAE (Figure A4); the kinetic of hydrolysis is comparable to that of DEX-avidin^17^ or HPMA^34^ when an ester bond is used to conjugate polymer and drug, importantly for the efficiency of the delivery system the hydrolysis appears complete (Figure 6) thus freeing all drug molecules loaded to PBAE. In this context, our results seem to suggest the critical role played by the type of bond used to conjugate polymer and drug over the type of polymer. It is has also been shown that DEX conjugation through a hydrazone, instead of an ester bond, results in a slower kinetics of drug release^17^, ^34^; consequently allowing the possibility of engineering slow and fast release systems. Nevertheless, when steroids are entrapped in hydrophobic system (micelle or liposome) the release is generally ineffective with the majority of drug remaining entrapped.^34^

The half-life of drugs in joint is about few hours,^9^, ^43^ thus “wash out” from cartilage is known to reduce treatment efficacy. For this reason it is important that a new delivery system for cartilage not only improves uptake but also retention^7^, ^9^; we have shown that DEX could be retained in cartilage through the use of PBAE-DEX for longer periods of time than when a commercial DEX phosphate solution is used (Figure 4). Because the cleavage of DEX from PBAE-DEX occurred in few hours, our drug retention results are likely to depend on the hydrolysis reaction kinetic; the possibility of tuning the pace of drug release through a different conjugation bond would allow enhancing retention further.

**Figure 5:**
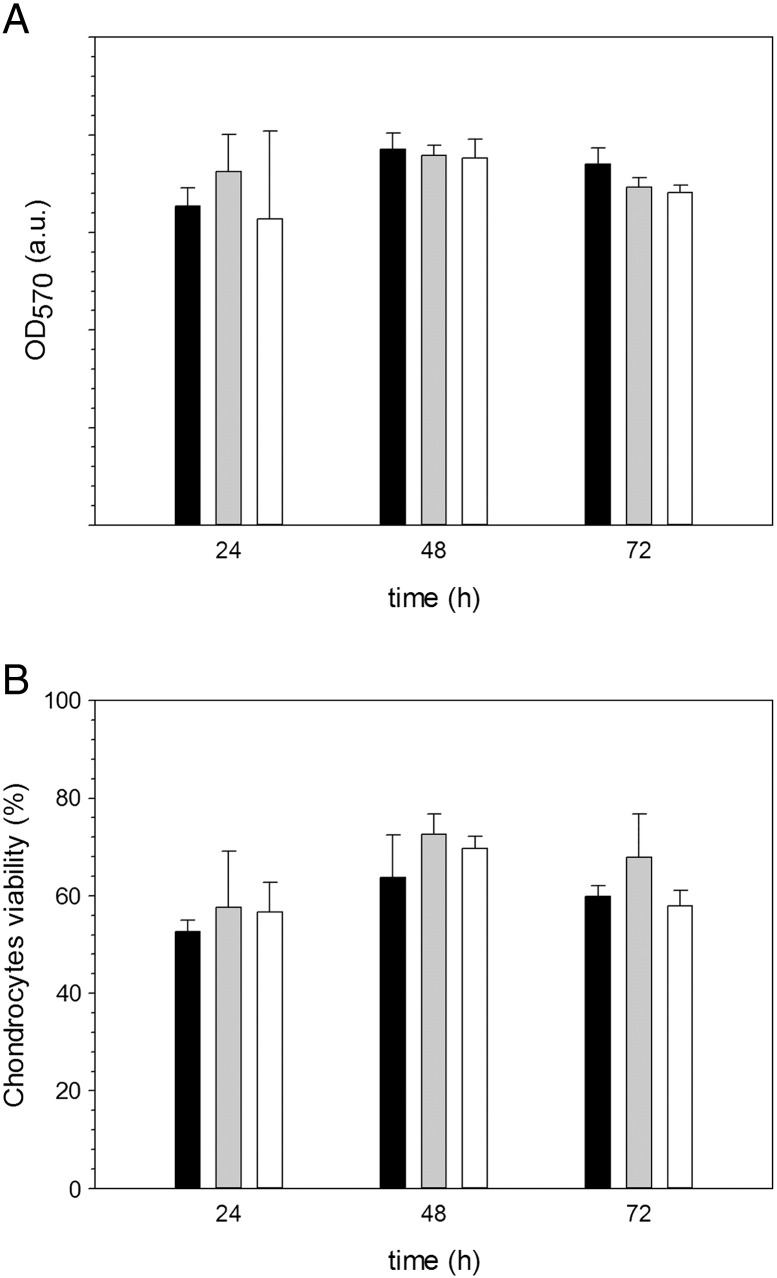
Viability of chondrocytes exposed to DEX-P (black columns), A1-DEX (gray columns) and A2- DEX (white columns) assessed through MTT **(A)** and (LDH) assay.

**Figure 6:**
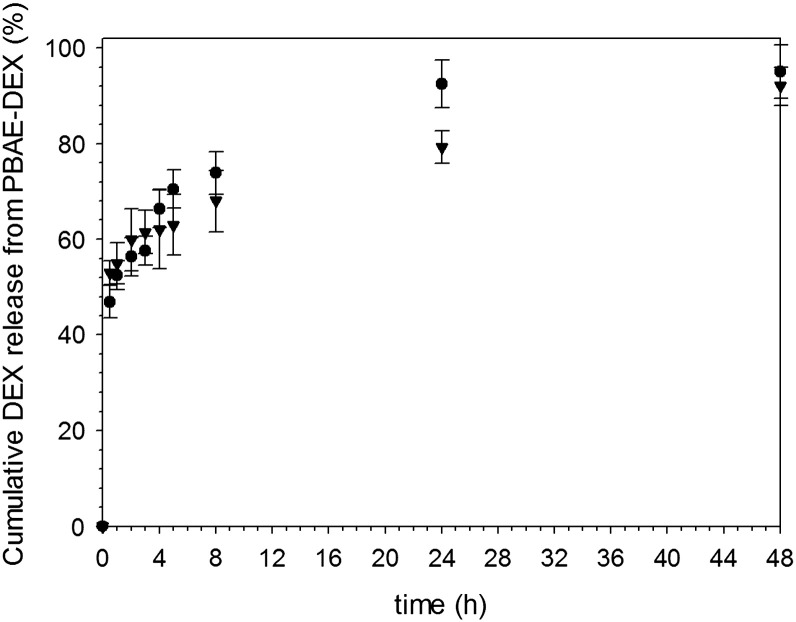
In vitro DEX release profile from PBAE-DEX. ● A1-DEX ▼ A2-DEX.

**Figure 7:**
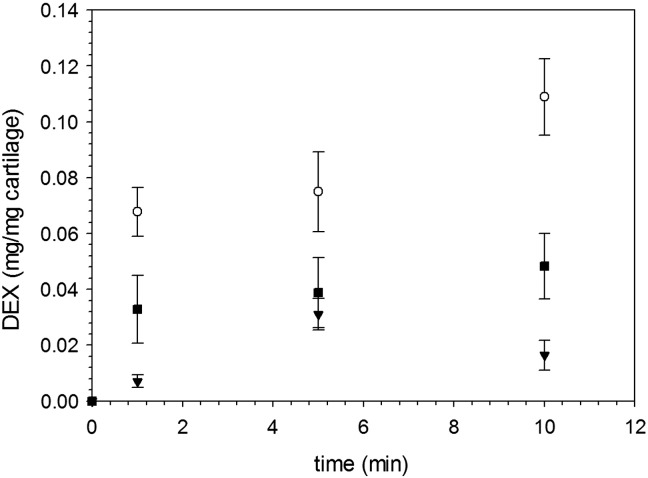
Comparison of DEX uptake in normal cartilage using PBAE mixed with DEX-P. ■ A1-DEX ▼ A2-DEX ◯ DEX-P.

Viability of chondrocytes was determined using two independent enzyme assays; MTT is based on mitochondrial activity whilst LDH is based on the relative concentration of lactate dehydrogenase in the media and inside the cells. We chose to use two tests as single assay could be inconclusive; for example a reduced metabolic activity could be interpreted as a reduction of viable cells if only MTT was employed.^44^, ^45^ Both tests revealed that chondrocytes were not affected by the presence of PBAE in the media (Figure 5) such providing essential information for future medical applications of the drug delivery system proposed in this work. This result was expected as PBAEs are known to be cytocompatible (a consequence of the easy hydrolyzation and biological compatibility of the degradation products) but gathering such experimental evidence was critical; as chondrocytes had not been exposed to PBAE yet. Moreover, our data clearly demonstrate that conjugation between DEX and PBAE is essential for enhancing drug uptake in cartilage (Figure 7).

One of the greatest advantages of PBAE over other molecules such as avidin is their relative low cost resulting from inexpensive monomers and ease of preparation, additionally the diffusion coefficient of PBAE in cartilages are 2–3 folds greater than avidin.^7^ Despite the large amount of research carried out with PBAE, our work is the first demonstrating their efficacy in augmenting drug uptake in cartilage and also the first where a drug is directly linked to the polymer chain.

We demonstrated, using DEX as a model drug, that PBAE constitute an effective drug delivery system for cartilage based on the electrostatic attraction between GAG and PBAE. We employed two strategies to link DEX to PBAE, one was to form a covalent bond between drug and carrier and another was to electrostatically bind the two; our results revealed that the covalent bond was essential to achieve the goal of developing a targeted drug delivery system for cartilage. In GAG depleted samples, resembling early stage of OA, the reduced driving force for the PBAE penetration did not hinder the efficacy of the delivery system; on the contrary the efficacy is enhanced likely in virtue of the higher hydraulic permeability of such cartilage tissue. The improved targeted delivery and retention of the chosen drug to cartilage has the benefit of improving efficiency as a lower amount of drug will be needed to achieve the same concentration in the tissue; particularly for mono-articular diseases where intra-articular injection is a common therapeutic approach. We, therefore, foresee OA treatments based on PBAE to boost drug uptake and retention in cartilage, simultaneously reducing drug off-site effects in virtue of the specificity and efficacy of its targeting mechanism and the overall cost of the treatment as PBAEs are inexpensive.

## Figures and Tables

**Table 1:** Mean and standard deviation of A1 and A2 chemical–physical properties.

PBAE	Zeta pot. (mV)	Size (nm)	Mw (kDa)	Mn (kDa)
A1	+11.60 ± 0.30	286 ± 63	12 ± 3	7 ± 3
A2	+8.94 ± 0.74	153 ± 36	13 ± 3	7 ± 3

## Appendix A Supplementary data

Supplementary material reaction schemes, DEX recovery after tissue digestion, cartilage sample preparation for microscopy and DLS results for PBAE. Supplementary material
