# Supplementary material for: Poly-beta-amino-esters nano-vehicles based drug delivery system for cartilage
Source: Nanomedicine. 2017 Feb;13(2):539–48. doi: 10.1016/j.nano.2016.10.001 (PMC5339075; doi:10.1016/j.nano.2016.10.001)
Supplement: Supplementary material — reaction schemes, DEX recovery after tissue digestion, cartilage sample preparation for microscopy and DLS results for PBAE. [file mmc1.pdf]

Poly-beta-amino-esters Nano-vehicles based Drug Delivery System for Cartilage

by

Stefano Perni [1], Polina Prokopovich [1]

[1] School of Pharmacy and Pharmaceutical Sciences, Cardiff University, Cardiff, UK

Supplementary information

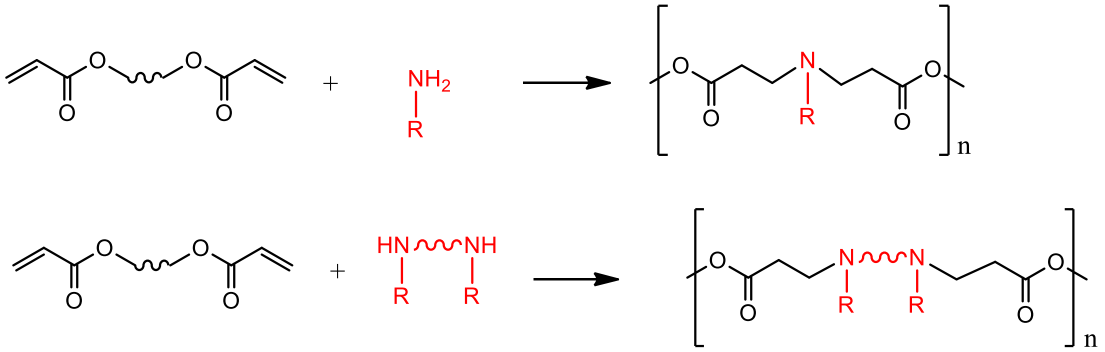

Figure A 1. Synthetic reaction and structure of PBAEs

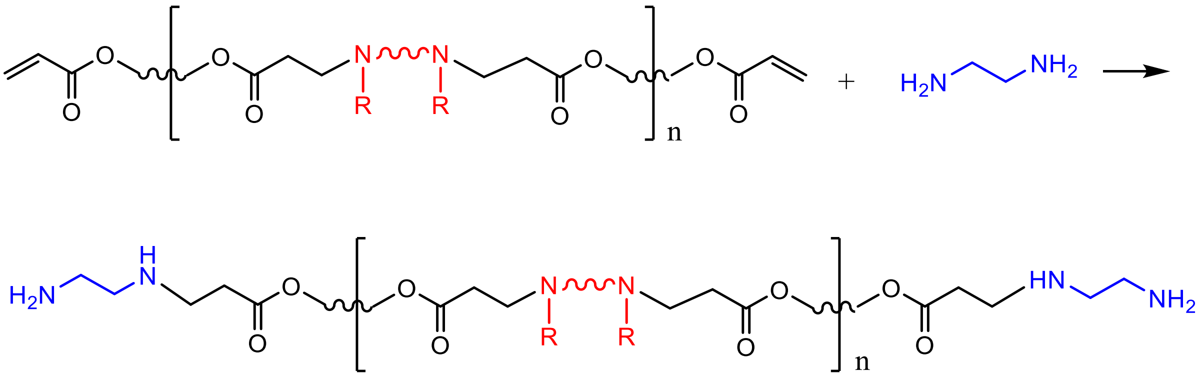

Figure A 2. Scheme of end-capping reaction of acrylate terminated PBAE with double amine compounds.

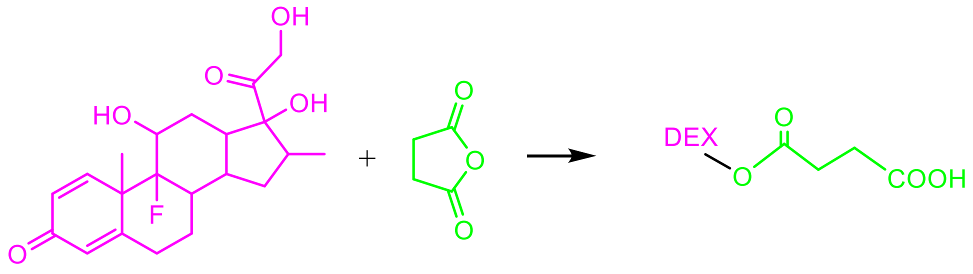

Figure A 3. Reaction scheme of Dexamethasone succinylation.

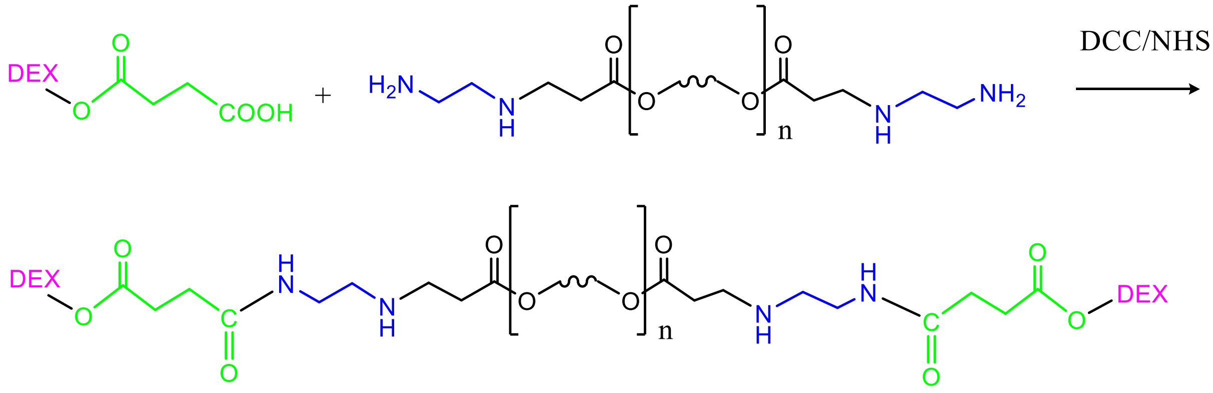

Figure A 4. Scheme of conjugation reaction between PBAE and succinylated dexamethasone.

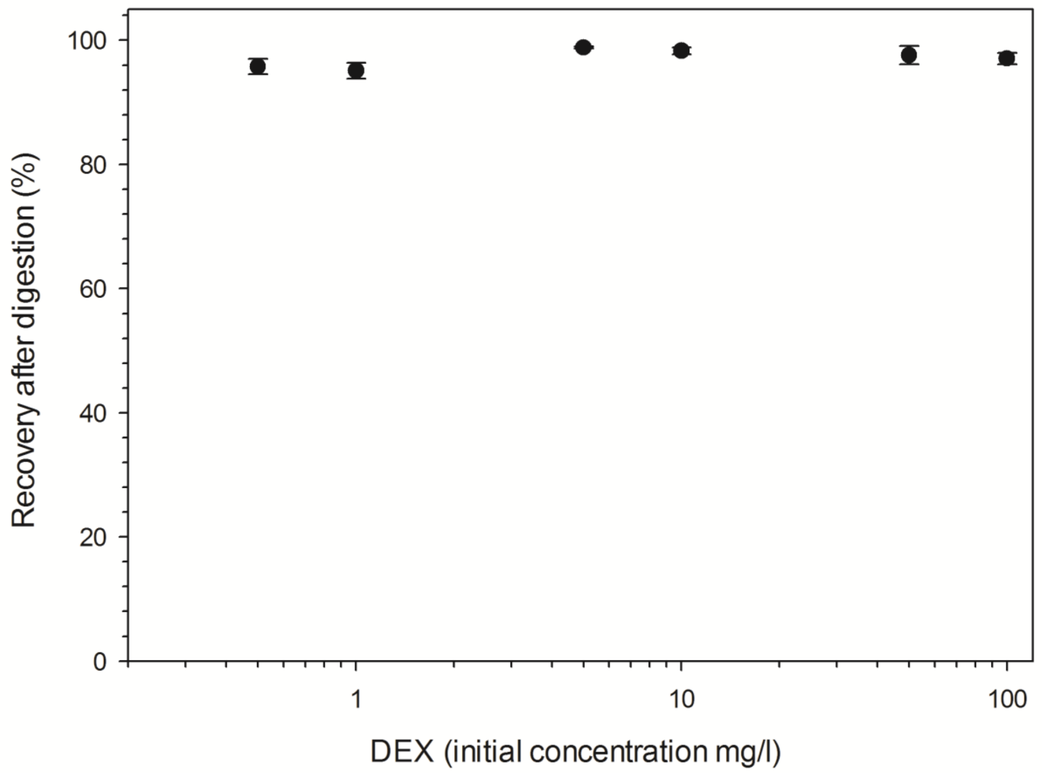

Figure A 5. DEX recovery after undergoing cartilage tissue digestion.

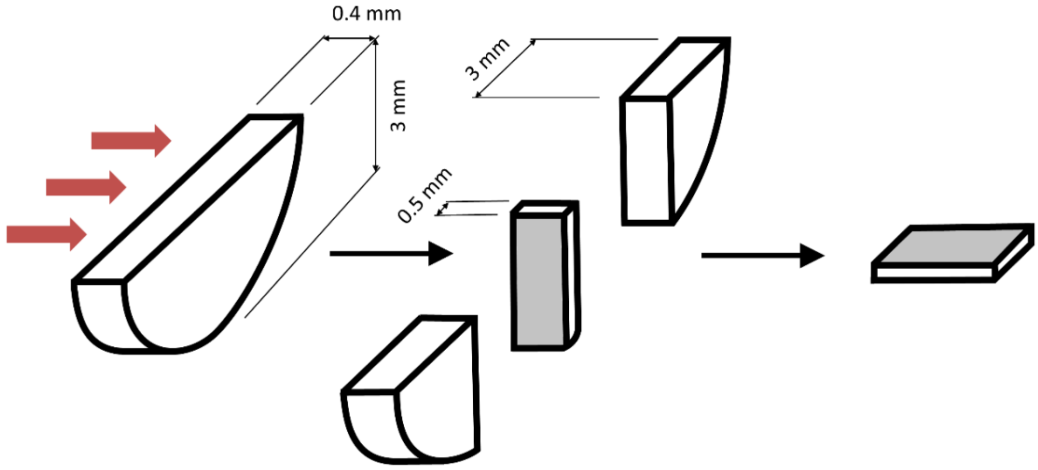

Figure A 6. Schematic description of the steps for the cartilage samples sectioning for microscopy imaging. Red arrows represent direction of PBAE flow and the surface imaged through epifluorescent microscopy is colored in gray.

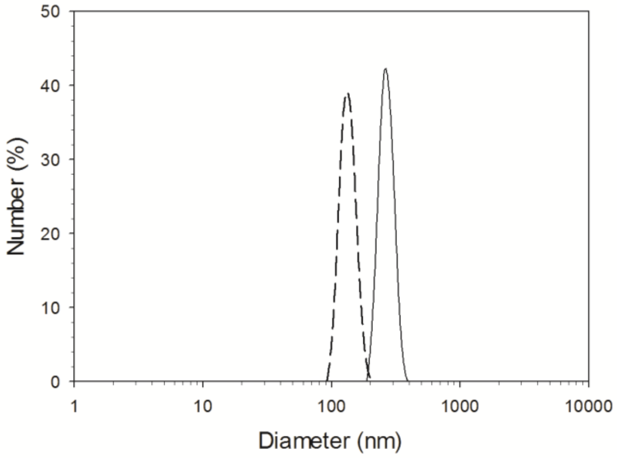

Figure A 7. Examples of DLS plots for A1 (solid line) and A2 (dashed line).
